# Supplementary material for: Studies in Rats of Combined Muscle and Liver Perfusion and of Muscle Extract Indicate That Contractions Release a Muscle Hormone Directly Enhancing Hepatic Glycogenolysis
Source: J Pers Med. 2022 May 20;12(5):837. doi: 10.3390/jpm12050837 (PMC9145889; doi:10.3390/jpm12050837)
Supplement: Supplementary file 1 [file jpm-12-00837-s001.zip › jpm-1694857-supplementary.pdf]

Supplementary Materials:

Figure S1: Hindquarter and liver combined perfusion.

Figure S2: Hindquarter and liver combined perfusion (close up).

Figure S3: Effect of flow in hindquarter and liver perfused in series.

Figure S4: Plasma glucose response to muscle extract injection in rats.

Table S1: Muscle glycogen concentrations (mmol glucose · kg wet tissue^{-1}).

Table S2: Perfusate pH and gas tensions.

Table S3: Perfusate concentrations of glucose and lactate.

Figure S1. Hindquarter and liver combined perfusion

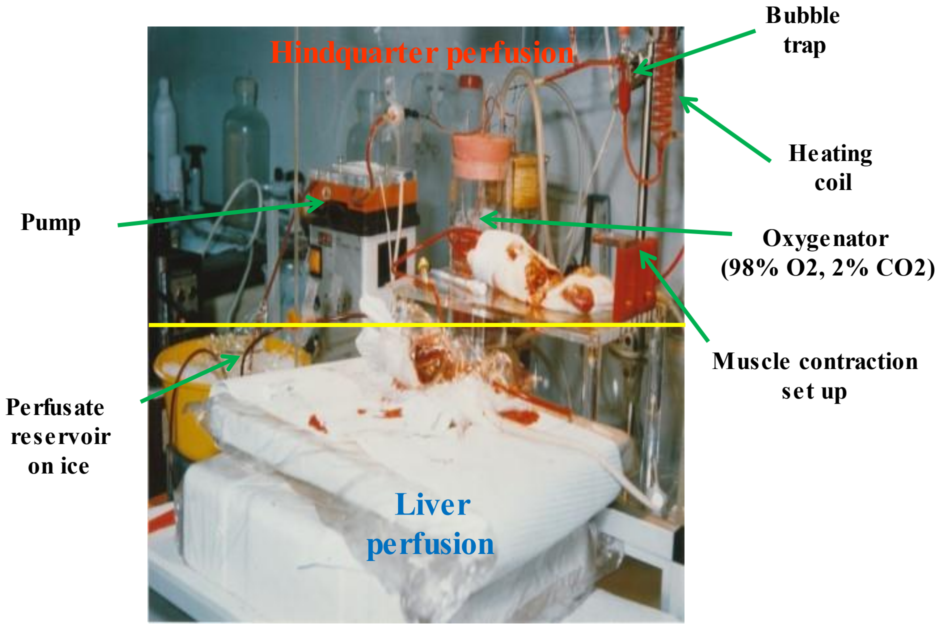

Experimental set up.

Figure S2. Hindquarter and liver combined perfusion (close up)

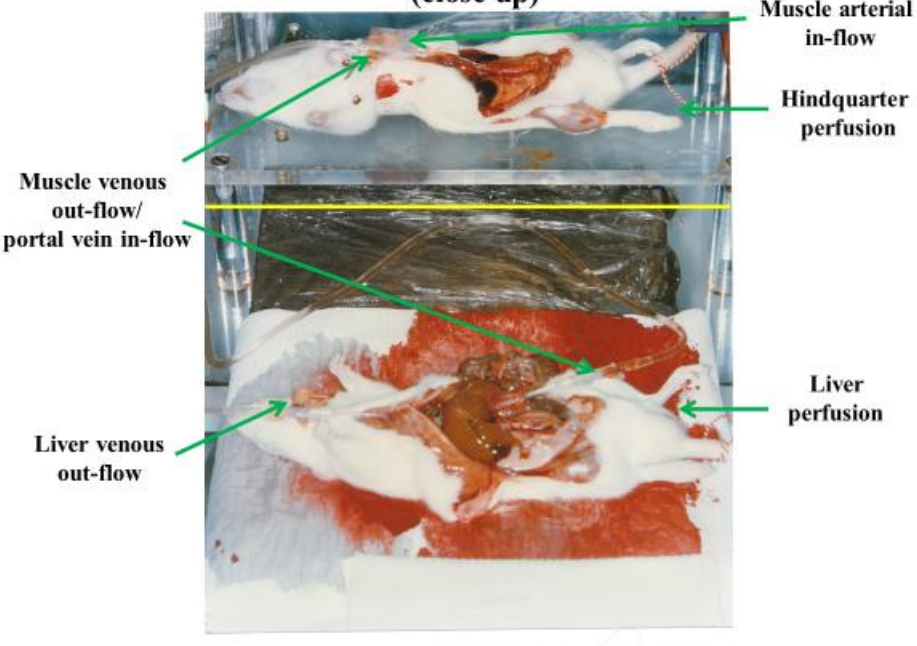

Experimental set up. Only for the close up photo, rats were perfused with cell-free medium. Otherwise, medium for combined muscle and liver perfusions included erythrocytes.

Figure S3. Effect of flow in hindquarter and liver perfused in series

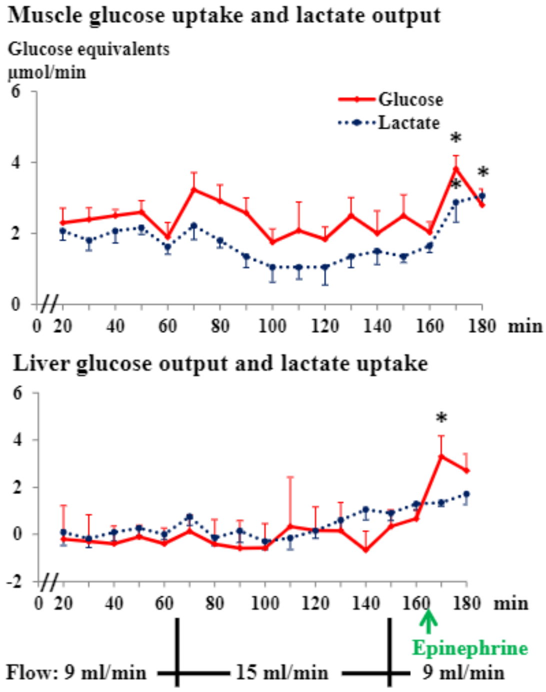

At min 150, flow was reduced to 9 ml/min and 10 min later epinephrine was added to a concentration of 20 nM in cell free perfusate. Values are means ± SE in 5 rats. * significantly different from basal values, $p<0.05$.

Figure S4. Plasma glucose response to muscle extract injection in rats

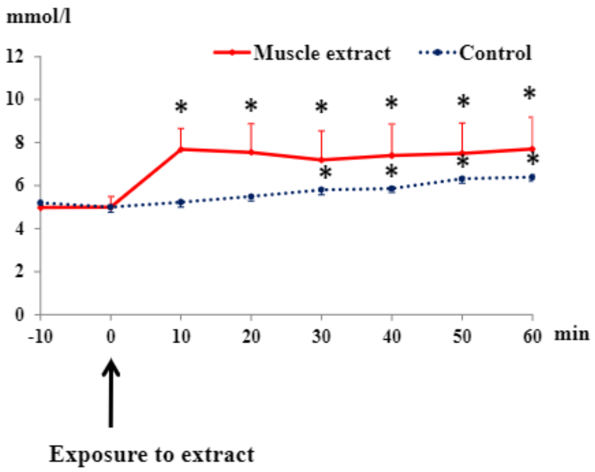

Data are means ± SE for groups of 7 rats. * significantly different from basal

values, $p<0.05$. The data shown for control rats represent responses to intraarterial injection of extract of epididymal fat, but identical responses were seen upon 0.9% NaCl injection.

Table S1. Muscle glycogen concentrations (mmol glucose · kg wet tissue^{-1}).

	White gastroc	Red gastroc	Soleus
Rest (5)	37±3	33±4	22±3
End of 2-legged contractions (7)	9±1*	8±1*	17±1*

Number of rats shown in parentheses. Values represent average of values from both legs and are means±SE. * significantly different from values at rest, $p<0.05$.

Table S2. Perfusate pH and gas tensions

			pH	PCO2, Torr	PO2, Torr	SAT(O2), %
One-legged Contraction (6)	Basal	A	7.40±0.01	40±2	438±21	100±0
		V	7.37±0.01	44±2	135±20	97±1
		L	7.32±0.02	52±4	46±7	70±6
	Contraction	A	7.32±0.01	49±3	420±15	100±0
		V	7.26±0.01	58±3	80±3	93±1
		L	7.25±0.01	61±3	50±4	76±4
Two-legged Contraction (7)	Basal	A	7.40±0.00	38±0	458±13	100±0
		V	7.35±0.01	46±1	79±2	95±0
		L	7.32±0.01	51±1	55±10	78±4
	Contraction	A	7.32±0.01	46±1	440±13	100±0
		V	7.24±0.01	58±1	75±1	91±1
		L	7.24±0.01	61±2	51±2	78±2
Flow increase (6)	Basal	A	7.40±0.01	40±1	427±22	100±0
		V	7.34±0.02	46±2	107±12	97±1
		L	7.30±0.02	54±2	45±2	75±3
	High Flow	A	7.33±0.02	47±2	431±18	100±0
		V	7.28±0.03	54±4	146±13	98±1
		L	7.26±0.03	58±4	59±2	85±2
Lactic acid Infusion (7)	Basal	A	7.47±0.01	35±1	584±21	100±0
		V	7.41±0.01	38±1	73±7	93±2
	Lactate	A	7.40±0.02	36±1	581±29	100±0
		V	7.38±0.02	39±0	196±36	98±1
Muscle Extract Infusion (8)	Basal	A	7.48±0.02	30±1	556±15	
		V	7.43±0.02	34±2	306±13	
	Extract	A	7.47±0.01	28±1	587±7	
		V	7.39±0.01	34±1	229±20	

Values are means±SE with no. of rats in parentheses. Perfusate pH, Pco_2 and Po_2 and oxygen saturation (SAT) were measured in the basal state as well as 5 min after either start of contractions or increase in perfusate flow in hindquarter-liver perfusions, or after increase in lactic acid concentration in isolated liver perfusion, or after additions of tissue extracts to perfused livers. In hindquarter-liver perfusions, A and V represents, respectively, arterial and venous samples from hindquarters, and L represents sample from liver vein. In the rest of the experiments, A and V represents, respectively, portal and hepatic venous samples.

Table S3. Perfusate concentrations of glucose and lactate

Time	One-legged Contraction		Time	Two-legged Contraction	
	Glucose (mM)	Lactate (mM)		Glucose (mM)	Lactate (mM)
40' A	6.09±0.13	0.88±0.03	40' A	6.44±0.08	1.1±0.08
40' V	5.90±0.12	1.17±0.05	40' V	6.17±0.10	1.31±0.09
40' L	5.76±0.14	1.30±0.07	40' L	6.15±0.11	1.34±0.09
70' A	5.88±0.13	1.25±0.07	50' A	6.36±0.11	1.27±0.09
70' V	5.69±0.11	2.35±0.20	50' V	6.01±0.12	2.86±0.16
70' L	5.92±0.13	2.20±0.13	50' L	6.37±0.20	2.63±0.13
80' A	5.91±0.13	1.70±0.07	60' A	6.29±0.18	2.04±0.18
80' V	5.66±0.11	2.32±0.13	60' V	5.91±0.16	2.81±0.16
80' L	5.64±0.15	2.08±0.12	60' L	6.02±0.16	2.46±0.16
90' A	5.79±0.12	1.87±0.09	70' A	6.08±0.16	2.36±0.18
90' V	5.54±0.11	2.23±0.15	70' V	5.67±0.18	2.76±0.20
90' L	5.50±0.13	2.08±0.16	70' L	5.76±0.17	2.47±0.19
110' A	5.51±0.12	2.00±0.12	110' A	5.25±0.19	2.44±0.24
110' V	5.30±0.13	2.18±0.12	110' V	4.87±0.18	2.71±0.22
110' L	5.21±0.14	2.03±0.15	110' L	4.98±0.18	2.34±0.25

Concentrations of glucose and lactate in perfusate plasma in hindquarter artery (A) and vein (V, also portal vein) and in liver vein (L) during one-legged and two-legged muscle contractions. One-legged contractions were begun after 65 min and lasted 85 min. Two-legged contractions were begun after 45 min and lasted 60 min. Data are means ± SE from 6-7 rats in each group.
